# Antiviral activity of iridoid glycosides extracted from *Fructus Gardeniae* against influenza A virus by PACT-dependent suppression of viral RNA replication

**DOI:** 10.1038/s41598-020-58443-3

**Published:** 2020-02-05

**Authors:** Shanshan Guo, Lei Bao, Chun Li, Jing Sun, Ronghua Zhao, Xiaolan Cui

**Affiliations:** Institute of Chinese Materia Medica, China Academy of Chinese Medical Sciences, No.4 Yinghua East Road, Chaoyang District, Beijing, 100029 China

**Keywords:** Antivirals, Drug development

## Abstract

Epidemic and pandemic influenza A virus (IAV) poses a significant threat to human populations worldwide. Iridoid glycosides are principal bioactive components from the *Gardenia jasminoides* J. Ellis fruit that exhibit antiviral activity against several strains of IAV. In the present study, we evaluated the protective effect of *Fructus Gardeniae* iridoid glycoside extracts (IGEs) against IAV by cytopathogenic effect(CPE), MTT and a plaque formation assay *in vitro* and examined the reduction in the pulmonary index (PI), restoration of body weight, reduction in mortality and increases in survival time *in vivo*. As a host factor, PACT provides protection against the pathogenic influenza A virus by interacting with IAV polymerase and activating the IFN-I response. To verify the whether IGEs suppress IAV replication in a PACT-dependent manner, IAV RNA replication, expression of PACT and the phosphorylation of eIF2α in A549 cells were detected; the levels of IFNβ, PACT and PKR in mouse lung tissues were determined; and the activity of IAV polymerase was evaluated in PACT-compromised cells. The results indicated that IGEs sufficiently alleviated cell damage and suppressed IAV replication *in vitro*, protecting mice from IAV-induced injury and lethal IAV infection. These anti-IAV effects might be related to disrupted interplay between IVA polymerase and PACT and/or prevention of a PACT-dependent overactivated IFN-I antiviral response. Taken together, our findings reveal a new facet of the mechanisms by which IGEs fight the influenza A virus in a PACT-dependent manner.

## Introduction

Influenza viruses cause recurrent seasonal epidemics or pandemics of respiratory infection that lead to 3 million to 5 million cases of severe illness and 250,000 to 500,000 deaths worldwide every year^1,2^. Especially in infants, young children, pregnant women, the elderly, and patients with weakened immune systems, influenza is likely to cause serious complications; therefore, the World Health Organization (WHO) has identified influenza as a major public health problem^3,4^. Furthermore, emergence and spread of new high pathogenic influenza variants have posed a critical challenges to the global health and include swine-origin H1N1, H5N8, H9N2, and H7N9^5–8^. In China, a novel H7N9 avian influenza virus caused 5 nationwide outbreaks from January 2013 to June 2017^9^. It was estimated that the average risk of fatality associated with H7N9 infection is approximately 40%^10^, which is much higher than that of SARS(11%)^11^.

Although of viral neuraminidase (NA) inhibitors and M2 ion channel blockers are widely used for treatment^12^, some serious side effects and low efficacy have been reported, and the emergence of drug resistant strains is increasing^13,14^. Influenza RNA-dependent RNA polymerase (RdRP) which is composed of three subunits, PA, PB1 and PB2, is a very promising alternative target for novel anti-influenza drug discovery research because it is essential for the transcription and replication of virus genes and is conserved among different types of influenza viruses^15,16^. During transcription, RdRP synthesizes capped and polyadenylated mRNA using 5′ capped RNA primers. During replication, RdRP generates a complementary RNA (cRNA) replication intermediate, which serves as a template for the synthesis of new copies of viral RNA (vRNA)^17,18^.

Influenza virus polymerase requires a large number of host cell factors to engage in the viral RNA transcription and replication processes^19,20^. The interactions between influenza polymerase and host cell factors were identified by diverse methods, such as genome-wide RNA interference (RNAi) screens^21^, proteomic approaches^22^, and systematic immunoprecipitation analysis^23^. Host factor PACT is a double-stranded RNA-binding protein that is also known to be an activator of the cellular dsRNA-dependent protein kinase, PKR^24^. PACT is involved in the interaction between influenza A virus (IAV) and host cells, and it can substantially enhance RIG-I activation and phosphorylate eukaryotic translation initiation factor 2 alpha (eIF2α) to induce an IFN type I response, which is suppressed by the nonstructural influenza virus protein NS1^25,26^. Recently, researchers have emphasized that, during IAV infection, PACT exerts its antiviral activity primarily by interacting with and inhibiting IAV polymerase. It has been demonstrated that PACT is associated with PA, PB1, and PB2^27^. Compromising PACT in IAV-infected A549 cells led to the enhancement of vRNA transcription and replication and IFNβ production. In addition, vRNA replication was increased by knocking down PACT in both A549 cells and IFN-insufficient Vero cells^28^.

Iridoid glycosides are the principal bioactive components in *Fructus Gardeniae*, which has antiviral and anti-inflammatory characteristics and has been used as a general Chinese herbal medicine for centuries in several Asian counties^29^. It has been reported that geniposide, a main iridoid glycoside compound extracted from *Gardenia jasminoides* J. Ellis fruit, effectively blocked cell damage induced by the pandemic A/Jiangsu/1/2009 (H1N1) influenza virus and alleviated virus-induced acute lung injuries^30^. In our previous study, it was demonstrated that *Fructus Gardeniae* iridoid glycoside extracts (IGEs) exhibited antiviral effects against influenza A virus H1N1 and H3N2 subtypes *in vitro* and *in vivo*^31^. RNA replication of the influenza virus A/FM1/47 was inhibited by IGEs in a dose-dependent manner, but the underlying mechanism has not yet been deciphered.

In the present study, we first determined the protective effect of the *Fructus Gardeniae* iridoid glycosides extracts (IGEs) on the cells and mice infected by influenza A virus. Next, we investigated whether the IGEs could inhibit vRNA replication and host factor PACT activation by evaluating the levels of virus replication, protein expression of PACT and phosphorylation of eIF2α in A549 cells and the levels of IFNβ, PACT and PKR in mouse lung tissues. In addition, to assess whether IGEs inhibit influenza virus replication in PACT-dependent manner, we measured RNA polymerase activity of influenza virus in HEK-293T cells in which PACT protein expression was knocked down by siRNA.

## Results

### Anti-influenza activity of the IGEs *in vitro*

**Figure 1:**
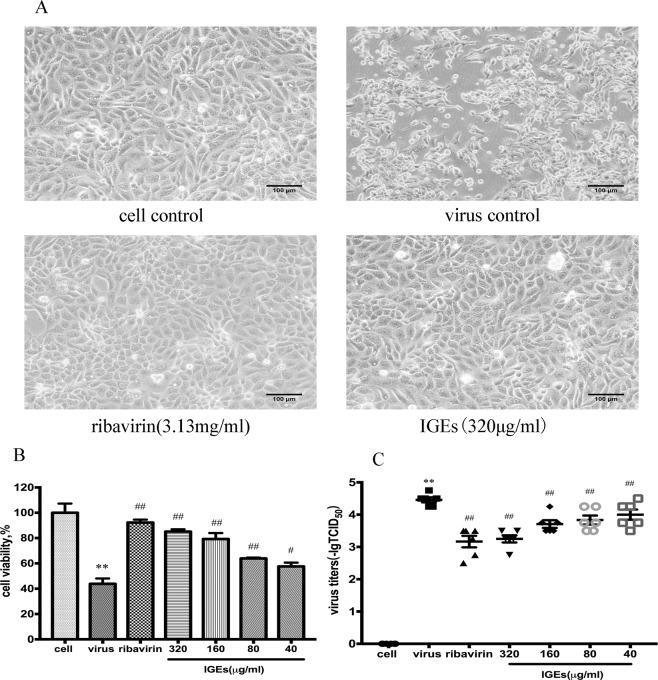
Antiviral activity of IGEs in Madin-Darby canine kidney (MDCK) cells infected with influenza A virus. (**A**) Cytopathic effect (CPE) observed in MDCK cells. There was no CPE in cell control group. Typical CPE characteristics, cell rounding and crimpling were observed in the virus control group, but were significantly alleviated in the ribavirin group and the IGEs treatment group. (**B**) MDCK cell viability of cells was evaluated by MTT assay. Each value was expressed as the mean ± SEM(n = 4). **P *<* *0.01* compared to the cell control group, ^*##*^*P* *<* *0.01* and ^*#*^*P* *<* *0.05* compared to the virus control group. (**C**) The value of virus titres for each group represented. Virus titres are shown as -lgTCID_50_ and expressed as the mean ± SEM (n = 6). **P *<* *0.01* compared to the cell control group, and ^*##*^*P* *<* *0.01* compared to the virus control group.

In the present study, the anti-influenza virus activity of the IGEs was measured by visualizing the CPE. MDCK cells showed a significant cytopathic effect (rounding and sloughing) at 48 h post infection, and the IGEs obviously inhibited the influenza virus A/FM1/47 (Fig. 1A).

To evaluate the protective effect of the IGEs on the MDCK cells induced by influenza virus, cell viability was further examined by MTT assay. Moreover, the MDCK cell virus titre was analysed by plaque formation assay. In the virus control group, cell viability was dramatically decreased, to 43.85%. IGEs treatment significantly increased the cell viability, to 85.08%, 79.26%, 63.92% and 57.60%, at concentrations of 320, 160, 80 and 40 μg/ml, respectively (Fig. 1B). Virus titres of the MDCK cells infected with influenza virus were markedly decreased by IGEs treatments (320, 160, 80 and 40 μg/ml) in a dose-dependent manner (Fig. 1C). The findings indicated that the influenza virus A/FM/1/47 was sensitive to IGEs treatment *in vitro*.

### IGEs inhibited the influenza-related high pulmonary index (PI) in mice

**Figure 2:**
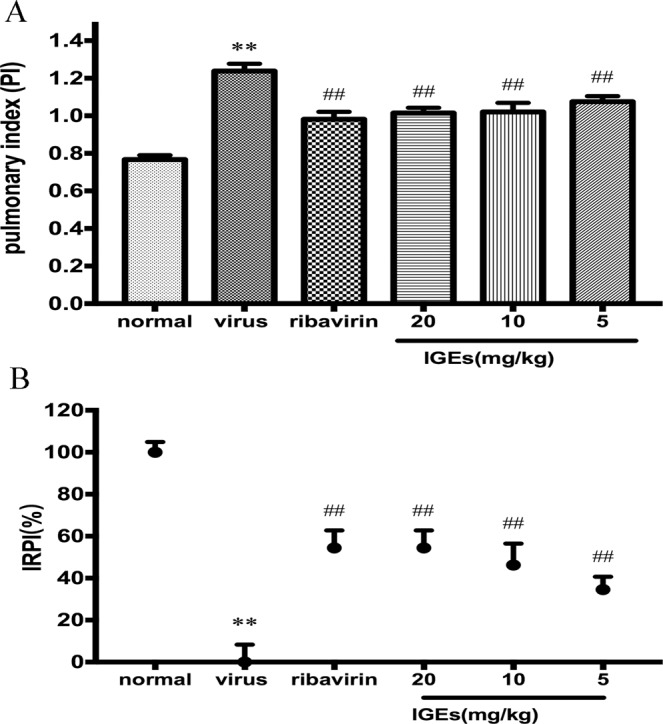
Inhibitory effect of the IGEs on the PI in an influenza mouse model. PI was expressed as the mean ± SEM(n = 10). **P *<* *0.01* compared to the cell control group, and ^*##*^*p* < *0.01* compared to the virus control group. B. IRPI was expressed as the mean ± SEM (n = 10). **P *<* *0.01* compared to the cell control group, and ^*##*^*p* < *0.01* compared to the virus control group.

In the current study, the anti-influenza activity of the IGEs *in vivo* was measured using PI and IRPI. PI was calculated to assess lung oedema. Mice in the virus control group presented with an increased PI (1.24 ± 0.04) compared to that presented by the normal control group (0.77 ± 0.02). Compared with that of the virus control group, groups treated with IGEs at doses of 20, 10, or 5 mg/kg presented with significantly decreased dose-dependent PI (Fig. 2A). In addition, groups treated with IGEs showed substantially inhibited PI activity, with the rate of the pulmonary index (IRPI) decrease of 54.40%, 46.23%, and 34.55% at the 20, 10, and 5 mg/kg dose, respectively (Fig. 2B).

### IGEs treatment protected mice from lethal influenza challenge

**Figure 3:**
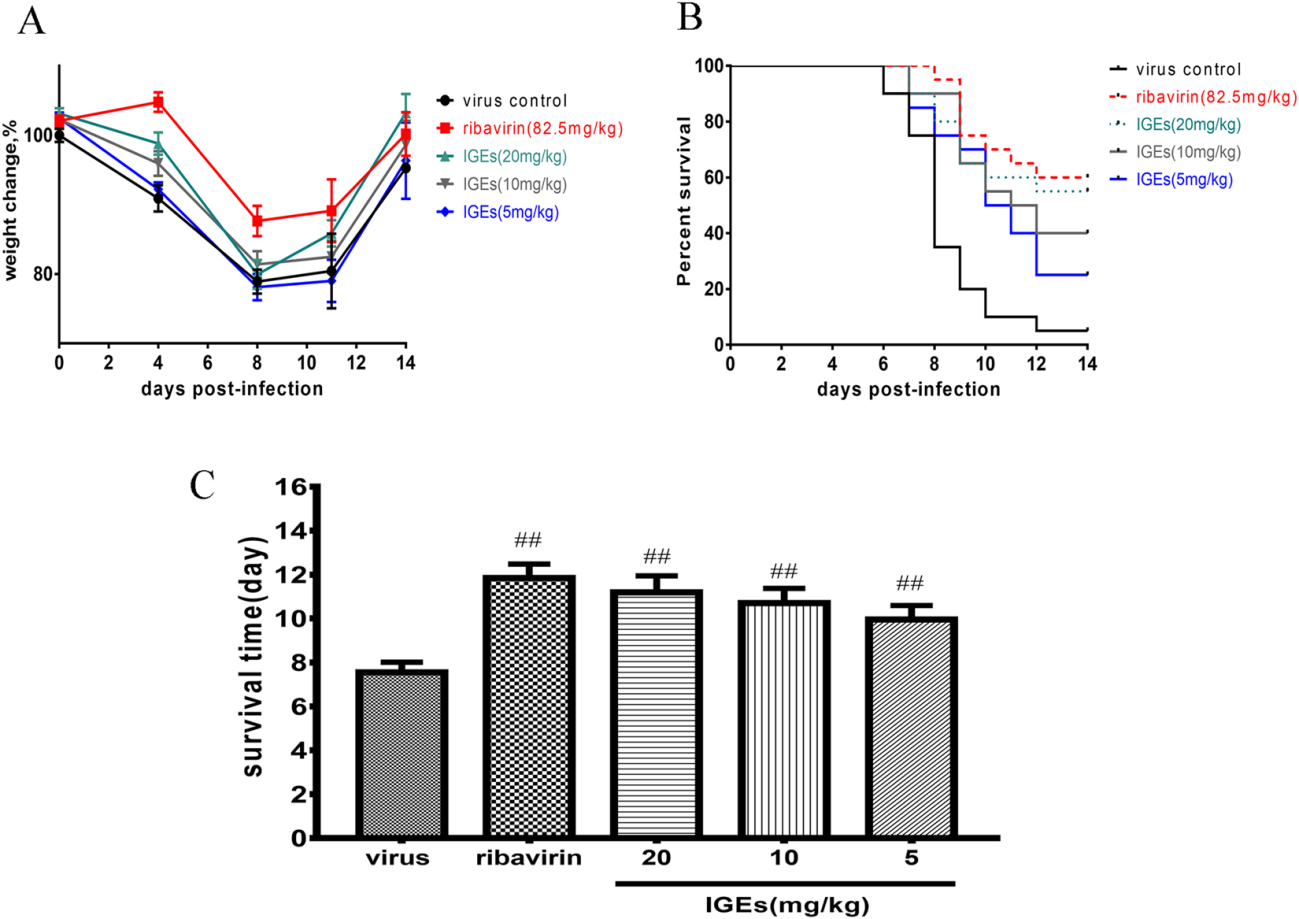
Protective effect of the IGEs against lethal IAV challenge to Balb/c mice. The mice were infected with intranasally with an influenza virus strain A/FM/1/47 solution and then treated with IGEs for 5 days. The body weight changes were determined by measurements taken 0, 4 8, 11, and 14 days post-infection, and the number of deaths in each group was recorded for 14 consecutive days (n = 20). (**A**) Body weight change curves for the 14 consecutive days. (**B**) Survival rate of the IAV- infected mice treated with IGEs (20, 10, 5 mg/kg) for 14 consecutive days. (**C**) IGEs treatment increased the survival time (days) of mice in a dose-dependent manner, ^*##*^*p* < *0.01* compared to the virus control group.

To evaluate the protective efficacy of IGEs against lethal influenza challenge, the change in body weight, reduction in mortality and prolonged survival time were estimated for the Balb/c mice. In the virus control group, the weight of the mice had mildly increased at 4 days post-infection, while at 8 days post-infection, the weight of mice had decreased to its minimum value. From 11 to 14 days post-infection, the weight of the mice visibly increased. IGEs treatment restored the body weight loss at 4, 8, 11, and 14 days post-infection (Fig. 3A).

Fourteen days after infection, 19 of the 20 mice in virus control group died, and the mortality was 95%. The mortality was significantly decreased to 45%, 60% and 75% by IGEs treatment at doses of 20, 10 and 5 mg/kg for the mice in the other groups compared to those in the virus control group. Moreover, IGEs treatment protected 11/20, 8/20, and 5/20 mice (55%, 40%, and 25%) from death at doses of 20, 10, and 5 mg/kg, respectively (Fig. 3B). In addition, IGEs treatment (20, 10 and 5 mg/kg) dramatically increased the survival time of the mice by 11.2, 10.7, 9.95 days compared to the survival time for the mice in the virus control group (Fig. 3C).

### IGEs inhibited influenza A virus replication

**Figure 4:**
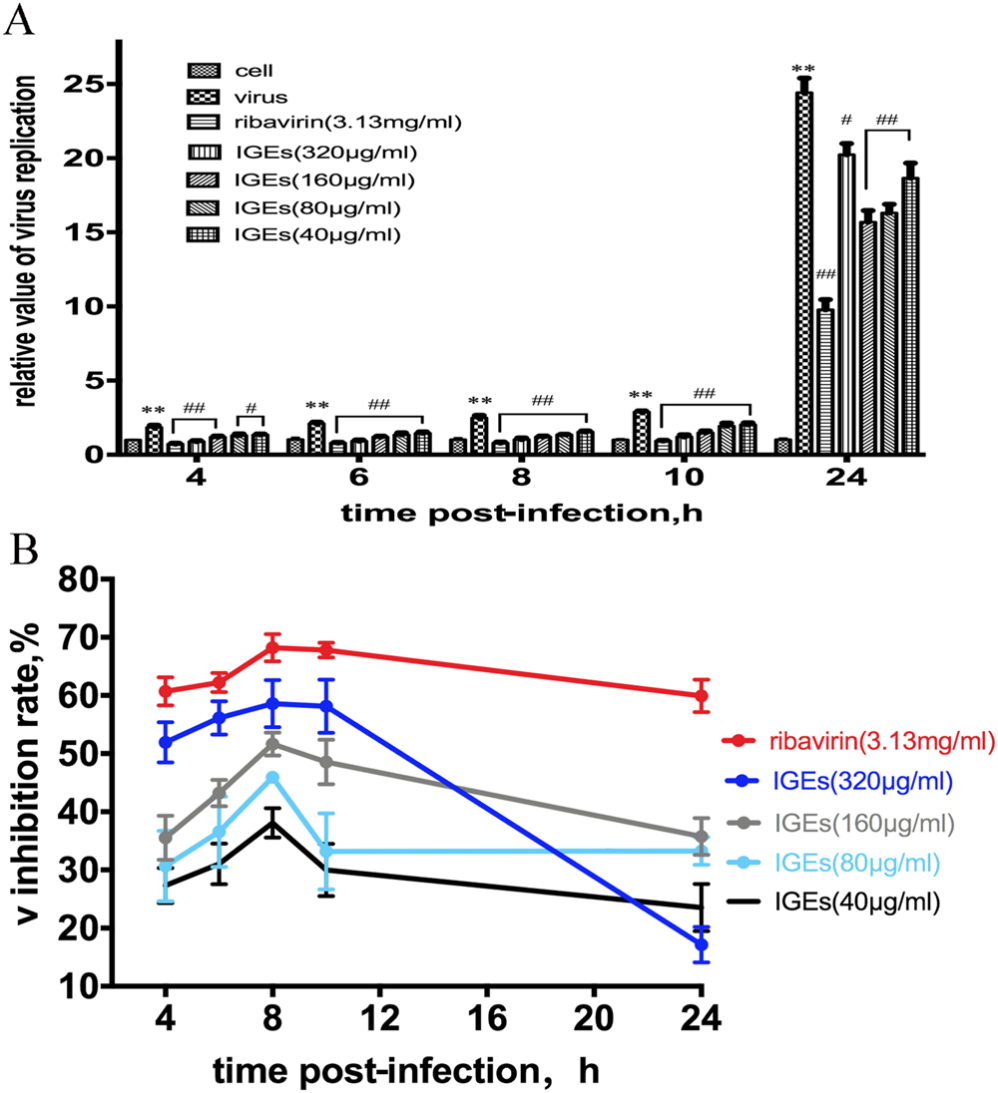
Inhibition of influenza virus replication by IGEs treatment of MDCK cells. The extent of IAV replication was detected by qRT-PCR at 4, 6, 8, 10, and 24 h post- infection. (**A**). Relative values of influenza virus replication were expressed as the mean ± SEM (n = 4). ^**^P *<* *0.01* compared to the cell control group, ^*##*^*P* *<* *0.01* and ^*#*^*P* *<* *0.05* compared to the virus control group. (**B**) The inhibitory rate of virus replication is expressed as the mean ± SEM (n = 4).

To explore the inhibitory effect of IGEs on influenza virus replication, the relative value of virus replication in the MDCK cells was analysed at 4, 6, 8, 10, and 24 h post-infection. The virus replication increased rapidly from 10 to 24 h post-infection. IGEs notably inhibited the replication of influenza virus A/FM1/47 in a dose-dependent manner at the five time points post-infection (Fig. 4A). IGEs showed the greatest inhibitory effect on virus replication 8 h post-infection, with virus inhibition rates of 58.58%, 51.67%, 45.94% and 38.08% at concentrations of 320, 160, 80 and 40μg/ml, respectively (Fig. 4B). The results demonstrated that IGEs significantly suppressed virus replication in the MDCK cells.

### Effect of IGEs on IFNβ production and PACT and PKR expression levels

**Figure 5:**
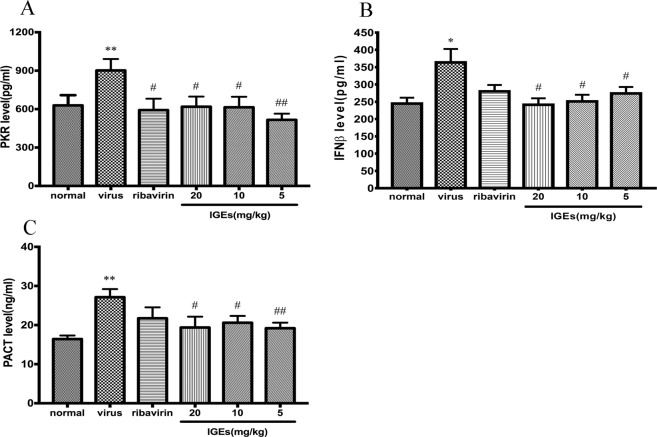
Effect of IGEs on IFNβ, PACT and PKR levels in lung tissues of ICR mice infected with IAV. Values are expressed as the mean ± SEM (n = 10), **p *<* 0.01, *p *<* *0.05* compared to the normal control group, and ^*##*^*p* *<* *0.01* and ^*#*^*p* *<* *0.05* compared to the virus control group. (**A**) The level of PKR. (**B**) The level of IFNβ. (**C**) The level of PACT.

PACT functions as an activator of RIG-I to facilitate the viral induction of type I IFNs. To verify the type I IFN response induced by IAV infection, the levels of IFNβ, PACT and PKR in the lung tissues were evaluated by ELISAs. Five days after the mice were inoculated with influenza virus A/FM1/47, the levels of IFNβ, PACT and PKR were remarkably more pronounced. Hence, IAV infection induced the type I IFN antiviral response that is activated by PACT and PKR. IGEs treatment significantly decreased IFNβ production at doses of 20, 10, and 5 mg/kg in a dose-dependent manner (Fig. 5A). Furthermore, as shown in Fig. 5B and Fig. 5C, the elevated levels of PACT and PKR in lung tissues of IAV-infected mice were depressed by the IGEs administered at doses of 20, 10, and 5 mg/kg.

### IGEs suppressed **PACT** expression **and** eIF2α phosphorylation activated by **IAV**

**Figure 6:**
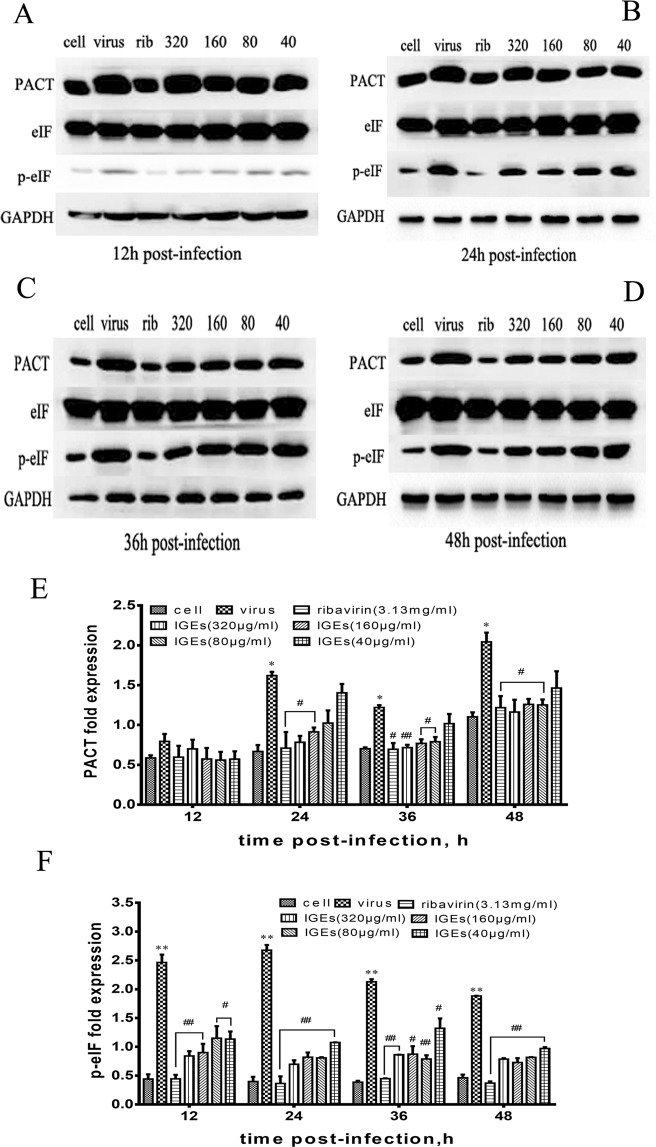
Effect of IGEs on PACT expression and eIF2α phosphorylation activated by IAV in A549 cells. Values are expressed as the mean ± SEM (n = 4), **p *<* *0.01* and *p *<* *0.05* compared to the normal control group, ^*##*^*p* *<* *0.01* and ^*#*^*p* *<* *0.05* compared to virus control group. (**A–D**) PACT expression and eIF2α phosphorylation were detected by western blotting in A549 cells at 12, 24, 36, and 48 h post infection. (**E**) The relative fold expression of PACT was calculated by the ratio of PACT to GAPDH. (**F**) The relative fold expression of p-eIF was calculated by the ratio of p-eIF to eIF.

Following activation by PACT, PKR phosphorylates its substrate, eIF2α. To assess the effect of the IGEs on PACT activation, PACT expression and eIF2α phosphorylation in A549 cells were detected by western blotting 12, 24, 36, and 48 h post-infection. PACT expression was markedly increased 24, 36, and 48 h post-infection. Accordingly, eIF2α phosphorylated 12, 24, 36, and 48 h after IAV infection. IGEs treatment apparently suppressed PACT expression in a dose-dependent manner at the three time points post-infection (Fig. 6A–E). Notably, eIF2α phosphorylation was inhibited by IGEs treatment at all time points (Fig. 6F). These results indicated that the overactivation of PACT induced by IAV was suppressed by IGEs treatment.

### IGEs inhibited the polymerase activity of IAV in PACT-deficient HEK-293T cells

**Figure 7:**
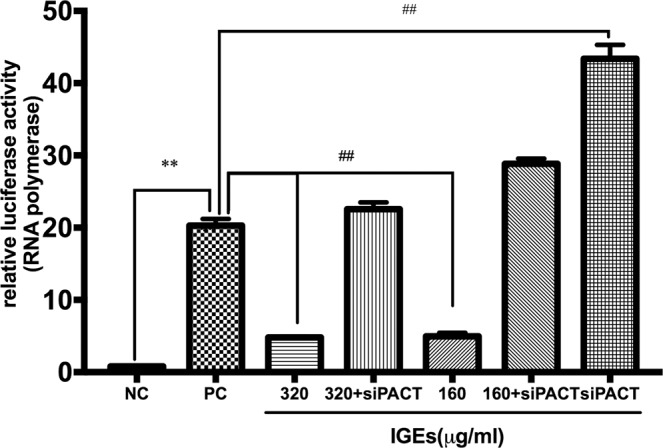
Relative luciferase activity of IAV polymerase in HEK-293T cells. Relative luciferase activity was detected using a dual-luciferase reporter assay system. Values are expressed as the mean ± SEM (n = 4), **p *<* *0.01* compared to the negative control group (NC), and ^*##*^*p* *<* *0.01* compared to the positive control group (PC).

As described above, we demonstrated that IGEs suppressed virus replication and PACT overactivation. To clarify whether the IGEs inhibited virus replication in a PACT-dependent manner, IAV polymerase activity was determined in the HEK-293T cells in which PACT protein expression was knocked down by siRNA. When PACT was depleted, RNA polymerase activity was more robust. Hence, virus replication that was catalysed by IAV polymerase was enhanced by the knockdown of PACT. In PACT-deficient HEK-293T cells, suppression of IAV polymerase activity was remarkably more robust at IGEs treatment doses of 320 and 160 μg/ml when PACT was not knocked down. The results suggested that the IGEs might inhibit virus replication in a PACT-dependent manner (Fig. 7).

## Discussion

Influenza virus causes severe respiratory illness annually, especially in elderly and immunocompromised individuals, leading to complications and hospitalization, and in some case, even death^32^. The currently used first-line drugs for the prevention and the treatment of influenza are neuraminidase inhibitors (NAIs), represented by oseltamivir^33^. However, the rapid emergence and spread of resistance to oseltamivir have created an urgent need for developing novel anti-influenza drugs with different mechanisms of action^34^. Some studies have reported that iridoid glycosides extracted from *Fructus Gardeniae* possess antiviral activity against influenza, including strains A/FM/1/47-MA, pandemic A/Jiangsu/1/2009, *in vitro and in vivo*^35,36^. Despite its great therapeutic potential, the IGEs ability to suppress virus replication or improve innate immune function was poorly understood.

In the present study, the results demonstrated that IGEs possess potent antiviral properties against influenza virus A/FM1/47. It could alleviate the cell damage and reduce plaque formation induced by the H1N1 subtype of influenza A virus at non-cytotoxic concentrations in a dose-dependent manner *in vitro*.

The pathogenic influenza A virus (H1N1) causes respiratory tract infection that develops into severe influenza pneumonia, leading to acute pulmonary injury (ALI)^37,38^ and acute respiratory distress syndrome (ARDS)^39,40^, even to death. In this study, we successfully established a mouse model of IAV pneumonia and death in which mice were infected with influenza virus A/FM1/47. In the mouse model of IAV pneumonia, IGEs exhibited an inhibitory effect on the pulmonary index, which indicated that the IGEs protected the lung tissues from influenza-induced injury. Of note, in the mouse death model, changes in body weight, reduced mortality and prolonged survival time suggested that IGEs treatment sufficiently protected and rescued mice from lethal IAV infection.

To verify whether IGEs inhibited virus replication, the expression of the influenza M1 gene in A549 cells was determined by quantitative real-time PCR. The results showed that virus replication in the A549 cells increased significantly 4, 6, 8, 10, and 24 h post-infection. In particular, the relative value indicating virus replication was sharply increased 24 h post-infection, and the value of the virus control group cells was more than 20-fold that of the control group cells. IGEs administration significantly suppressed influenza virus replication at all five time points in a dose-dependent manner. The results indicated that, during the IAV lifecycle, the IGEs were capable of inhibiting virus replication and propagation *in vitro*. To determine whether the IGEs affected innate immune defence against IAV, the levels of IFNβ, PACT and PKR in the lung tissues of IAV-infected mice were assessed.

Type I interferon (IFN-I) is a key component of the innate antiviral response, and constitutes the first line of host defence against influenza virus infection^41–43^. The activation of the IFN-I response requires PACT, which interacts with RNA polymerase, and nonstructural proteins NS1 and NS2 of influenza A virus^44^. Influenza virus replication depends on the interaction of virus-encoded proteins with host factors, which regulate pivotal aspects of cell survival; virus recognition, replication and pathogenicity; and host defence^45,46^. It has been reported that PACT is a crucial antiviral host factor that mediates the activation of the dsRNA-dependent kinase PKR, and stimulates substantial RIG-I activation to induce IFN-I production^47^. When PACT was co-expressed with RIG-I, the IFNβ promoter activity induced by IAV RNP complex was most robust^48^.

The outcome of IAV infection depends on the intricate interactions between the virus and the host. When IAV prevails, the antiviral activity of IFNs can insufficiently inhibit IAV replication, and the accumulation of vRNA and RNP results in overactivation of IFNs, which might lead to the development of pathological inflammation. In contrast, when IFNs prevail, IAV replication is suppressed and eventually cleared^49,50^. In the current study, IFNβ, PKR and PACT levels were remarkably elevated in the lung tissues of mice infected with IAV. The results indicated that IAV infection induced an IFN-I response in the mice and that PACT enhanced the activation of the PKR and IFN response, findings consistent with those from a recently reported study^51,52^. IGEs treatment at doses of 20, 10, 5 mg/kg significantly decreased the IFNβ production and depressed the elevated levels of PACT and PKR in lung tissues of the IAV-infected mice. Our findings revealed that IGEs might protect the IAV-infected mice from pneumonia and death by inhibiting the overactivation of the IFN response induced by PACT.

In response to stress signals induced by viral infection, four mammalian serine-threonine kinases phosphorylate the eukaryotic protein translation initiation factor 2α (eIF2α), including HRI, GCN2, PERK and PKR^53^. Among these kinases, IFN-I-induced PKR plays a crucial role in the anti-viral innate immune response against the influenza A virus. PACT-dependent PKR activation leads to phosphorylation of eIF2α, which results in the arrest of translation of both cellular and viral mRNAs, which is an efficient way to suppress virus replication^54,55^.

To verify the activation of PACT and eIF2α induced by IAV infection, we measured PACT expression and eIF2α phosphorylation in infected A549 cells 12, 24, 36, and 48 h post-infection. In the current study, PACT expression in the A549 cells increased significantly 24, 36, and 48 h after IAV infection. In addition, the phosphorylation of eIF2α in the A549 cells was dramatically increased 24, 36, and 48 h after IAV infection. The results demonstrated that IAV infection led to a PACT response and activated eIF2α phosphorylation, findings in general agreement with a previous report^56^. Furthermore, IGEs treatment markedly inhibited PACT expression in a dose-dependent manner at the three measurement time points post- infection. Interestingly, eIF2α phosphorylation was significantly suppressed by IGEs treatment at four time points. These results indicated that the overactivation of PACT and eIF2α phosphorylation induced by IAV was suppressed by IGEs treatment, which provides evidence that IGEs exert anti-IAV activity by inhibiting the overactivation of the PACT-dependent innate immune response.

PACT has been identified as an antiviral host factor that interacts with IAV polymerase. The interaction between PACT and IAV polymerase plays a pivotal role in the outcome of virus infection and antiviral immune response. It has been demonstrated that PACT-dependent activation of IFNβ production was inhibited by all three subunits of IAV polymerase^57,58^. In an RNP reconstitution assay, knocking down PACT in IAV-infected A549 cells enhanced vRNA production and the subsequent IFN response^59^. Of interest, there might be interplay between PACT and other viral molecules, such as the herpes simplex virus type 1 (HSV-1) Us11 protein, Middle East respiratory syndrome coronavirus (MERS-CoV) 4a and the nucleocapsid of the Ebola virus (EBOV) VP35, mouse hepatitis virus (MHV) N proteins and influenza A virus NS1^60–63^. Hence, PACT might be a common target to counteract innate immunity response induced by different viruses with the potential to be developed into a new broad-spectrum antiviral drug.

To further investigate whether IGEs suppresses IAV replication in a PACT-dependent manner, PACT was compromised by siRNA in HEK-293T cells that had been co-transfected with four plasmids containing NP, PA, PB1 and PB2. According to the results of the dual-luciferase reporter gene assay, the HEK-293T cells transfected with only with pFlu-luc showed very low relative luciferase activity for the IAV RNA polymerase. In contrast, cells co-transfected with plasmids containing NP, PA, PB1, PB2 and Flu-luc showed relatively high RNA polymerase activity. Of note, our results indicated that knocking down PACT enhanced IAV polymerase activity, a finding that was consistent with that of a previous study. Hence, virus replication that was catalysed by IAV polymerase was enhanced by compromising the action of PACT, which was identified as an antiviral host factor. In addition, IGEs treatment at doses of 320 and 160 μg/ml showed a relatively weak suppressive effect on IAV polymerase activity in the PACT-knockdown HEK-293T cells. Collectively, the data from the current study demonstrated that IGEs treatment might inhibit IAV replication in a PACT-dependent manner.

In conclusion, our research indicated that IGEs sufficiently alleviated cell damage and suppressed IAV replication *in vitro*, protected the lung tissues from IAV-induced injury in mice models of influenza-induced pneumonia and rescued mice from lethal IAV infection. These anti-IAV effects might be related to disrupted interplay between the IVA polymerase and the cellular double-stranded RNA-binding protein PACT, which prevents the overactivation of the PACT-dependent IFN-I antiviral response induced by IAV infection. Taken together, our findings reveal a new facet of the mechanism of IGEs action against the influenza A virus, for which it inhibited IAV replication in a PACT-dependent manner. The current study might pave the way for the development of new antiviral agents against IAV.

## Methods

### Biosafety statement

All experiments involving live H1N1 influenza viruses were conducted in an animal biosafety level 2 laboratory (ABSL-2) at the Institute of Chinese Materia Medica, China Academy of Chinese Medical Sciences.

### Sample preparation

First the powder of dried fruits (2 kg) was reflux-extracted twice with 70% ethanol, and then the extracts were combined and centrifuged. Next, the supernatant fluid was vacuum dried to remove traces of alcohol, and the concentrated solution was diluted with water to a mass concentration of 0.5 g/ml. The filtrate was passed through AB-8 macroporous resin and then successively eluted with water and 50% ethanol. The 50% ethanol eluted fractions were combined and dried as the extract sample. The sample was stored at 4°C and dissolved in double distilled water at the time of use. The purity of the geniposide was 39.4%, and the purity of the iridoid glycosides in the sample was > 90%, as detected by UV spectrophotometry(T6, Beijing General Instrument Co. LTD, Beijing, China).

### Virus and cells

Influenza A virus (H1N1) strain FM/1/47 was provided by the National Institute for Viral Disease Control and Prevention, Chinese Center for Disease Control and Prevention. The virus stock was prepared by infecting 10-day-old specific-pathogen-free embryonated chicken eggs (Merial Vital Laboratory Animal Technology Co., Ltd, Beijing, China) for 48 h at 35°C. The virus titre (expressed as 50% tissue culture infective dose, TCID_50_) was calculated to be 10 ^−4.5^ in the MDCK cells by the Reed-Muench method^64^.

MDCK, A549 and HEK-293T cells were purchased from the Cell Center, Institute of Basic Medical Sciences, Peking Union Medical College (Beijing, China) and cultivated in DMEM supplemented with 10% foetal bovine serum and 1% penicillin/streptomycin (all from Gibco).

### Animals

ICR mice(13–15 g) and Balb/c mice(18–22 g) were obtained from Vital River Laboratory Animal Technology Co., Ltd. in Beijing, China, and acclimated with standard feed and water in the following conditions (temperature: 20 ± 2°C, humidity: 50 ± 10%). All experiments were performed in compliance with the rules of the European Community Guidelines for the care and use of animals. The study was approved by the Ethics Committee at Institute of Chinese Materia Medica, China Academy of Chinese Medical Sciences.

### *In vitro* antiviral assay

MDCK cells were cultured in 96-well plates at a density of 5×10^5^ cells/ml, and exposed to the FM/1/47 strain of H1N1 influenza virus at 100 TCID_50_ for 1 h. Then, the infected cells were incubated for 48 h with serially diluted IGEs solutions, to final concentrations were of 320, 160, 80 and 40μg/ml. Ribavirin was used as a positive drug control, at a final concentration of 3.13 mg/ml. After incubation at 37 °C for 48 h, the infected cells showed a significant cytopathic effect (CPE), distinguished by rounding and sloughing. The inhibition effect of IGEs on the CPE induced by influenza A virus was determined by MTT assay. Cell viability was expressed as the percentage of the cell control group (100%). The virus titres were quantified by plaque formation assay and presented as the negative lg 50% tissue culture infectious dose (-lgTCID_50_)^65^.

### Animal infection and treatment

Normal control group, virus-infection control Sixty ICR mice (13–15 g), 30 males and 30 females, were randomly separated into six groups: group, ribavirin treatment group (82.5 mg/kg), and three IGEs treatment groups based on administered IGEs dose: 20, 10 and 5 mg/kg. All mice except those in the normal control group were infected intranasally with 40 μL of viral suspension of influenza virus/A/FM/1/47(H1N1) at a concentration of 15 times of median lethal dose (LD_50_). In the treatment groups, the inoculated mice were successively given daily doses of IGEs or ribavirin orally for 4 days beginning on the day of infection. Each mouse in the control group was given a volume of distilled water equal to that of the IGEs treatment volume. On day 5, all the mice were sacrificed, and the body weight (BW) and lung weight (LW) of each were determined to calculate the pulmonary index (PI) and the inhibitory rate of pulmonary index (IRPI). PI = [LW(g)/BW(g)]×100, IRPI = [(LW of virus control group-LW of treatment group)/(LW of virus control group-LW of normal control group)] ×100. The lung tissues were collected and subjected to an ELISA.

### Mouse model of death due to influenza virus infection

A total of 100 Balb/c mice were randomly distributed into five groups with 20 mice per group: the virus-infection control group, ribavirin treatment group, and three IGEs treatment groups. All the mice were infected with 45 μL of the influenza virus strain A/FM/1/47 solution at a concentration 8-fold greater than that of the LD_50_ by intranasal instillation. In the treatment groups, the inoculated mice were successively given IGEs orally at a dose of 20 ml/kg, 10 ml/kg or 5 ml/kg, or treated with 82.5 mg/kg ribavirin daily for 5 days at the beginning of the infection day. Each mouse in the control group received a volume of distilled water equal to the treatment volume. Body weight changes were determined at days 0, 4 8, 11, and 14 post-infection, and the survivals of the mice were recorded every day for 14 consecutive days. The protective effect against the lethal challenge was determined as increased body weight, reduction in mortality and prolonged survival time.

### Influenza virus replication levels detected by qRT-PCR

The levels of influenza virus A/FM/1/47 replication in MDCK cells were detected by quantitative real-time RT-PCR. Total cellular RNA was extracted 4, 6, 8, 10, and 24 h post-infection by TRIzol reagent (Invitrogen, USA) according to the manufacturer’s instructions. The QRT-PCR assay was performed using the one-step SYBR PrimeScript RT-PCR kit (TaKaRa, Japan) according to the manufacturer’s protocol (for the influenza virus A/FM/1/47 M protein genes the forward primer was 5′-ATCATTGGGATCTTGCACTT-3′, and the reverse primer was 5′-TCATAGACTCTGGCACTCCTT-3′, and for GAPDH, the forward primer was 5′-CCCACTCCTCCACCTTTGACG-3′, and the reverse primer was 5′-CACCACCCTGTTGCTGTAGCCA-3′). The transcript levels of influenza virus A/FM/1/47 and GAPDH were calculated by the ΔΔCT method. PCR was carried out using a quantitative PCR instrument (Thermo, USA) under the following conditions: 42 °C for 5 min, 95 °C for 10 sec followed by 40 cycles of 95 °C for 5 sec and 60 °C for 34 sec. The inhibitory rate of virus replication = 1 − (relative value of virus replication in the treatment group)/(relative value of virus replication in the virus control group).

### ELISAs for determining IFNβ, PACT and PKR levels

The levels of IFNβ, PACT and PKR in the mouse lung tissues were detected by enzyme-linked immunosorbent assay (Elisa) kits (96 T, Meilian Co., Ltd, Shanghai, China) following the manufacturer’s instructions.

### Western blot assay of PACT expression and phosphorylation of eIF2α

A549 cells were lysed and dissected in RIPA buffer containing protease inhibitor and protease phosphatase inhibitor at 2, 24, 36, and 48 h post-infection and proteins.

The total protein concentrations were determined using a BCA kit to ensure equal amounts of samples. Proteins were loaded on SDS-PAGE gels (10%) and then transferred onto a 0.45 μm nitrocellulose membrane. The membranes were incubated overnight at 4 °C with the primary antibodies against the following proteins: PACT (1:2000, Abcam, USA, eIF2α and GAPDH (1:2000, CST, USA), p-eIF2α, (1:500, CST, USA). Subsequently, the membranes were incubated with secondary goat anti-rabbit IgG antibody (1:4000) for 1.5 h at room temperature. The blots were developed by ECL, and the density of the bands was calculated by NIH ImageJ software.

### Dual-luciferase reporter gene assay

First, HEK-293T cells were transfected with PACT siRNA (forward primer 5′- GCAUGAAGACCAAGAACAGTT-3′ and reverse primer 5′- AUGUUCUUGGUCUUCAUGCTT-3′) for 12 h at 37° in 5% CO_2_. Next, four plasmids containing RdRP cDNA from influenza virus A/WSN/33 (H1N1) (pHW181-PB2, pHW182-PB1, pHW183-PA, and pHW185-NP) and pFlu-luc were co-transfected into HEK-293T cells using a Lipofectamine 3000 transfection kit (Invitrogen, USA) according to the method described in a previous study^66^. For the negative control group, only pFlu-luc was transfected into the cells. At 12 h post-transfection, the supernatant was discarded. Subsequently, two concentrations of IGEs (160 and 320 μg/ml) were added and incubated for 24 h at 37 °C in 5% CO_2_. Finally, the luciferase activity was detected using a dual-luciferase reporter assay system (Promega, USA) according to the manufacturer’s protocol. Luminescence intensity was read using a Thermo scientific™ Varioskan Flash reader (USA). The relative luciferase activity was calculated based on the ratio of the *Renilla* luciferase value to firefly luciferase value.

### Statistical analysis

Statistical analysis was performed using the GraphPad Prism 7.0 software system. All data are presented as the mean ± SEM (standard error of the mean). A χ^2^ test was used to analyse the reduction in mortality. Other results were analysed by one-way analysis of variance (ANOVA), and significant differences were determined by the Bonferroni Test. *P* values < 0.05 were considered significant.

## Data Availability

All data generated or analysed during this study are included in this published article.
